# A Comparative Evaluation of Nanohydroxyapatite-Enriched Hydrogen Peroxide Home Bleaching System on Color, Hardness and Microstructure of Dental Enamel

**DOI:** 10.3390/ma14113072

**Published:** 2021-06-04

**Authors:** Riccardo Monterubbianesi, Vincenzo Tosco, Tiziano Bellezze, Giampaolo Giuliani, Mutlu Özcan, Angelo Putignano, Giovanna Orsini

**Affiliations:** 1 Department of Clinical Sciences and Stomatology, Polytechnic University of Marche, 60126 Ancona, Italy; r.monterubbianesi@univpm.it (R.M.); v.tosco@pm.univpm.it (V.T.); a.putignano@univpm.it (A.P.); 2 Department of Materials, Environmental Sciences and Urban Planning, Polytechnic University of Marche, 60131 Ancona, Italy; t.bellezze@univpm.it (T.B.); g.p.giuliani@univpm.it (G.G.); 3 Center of Dental Medicine, Division of Dental Biomaterials, Clinic of Reconstructive Dentistry, University of Zurich, 8032 Zürich, Switzerland; mutlu.ozcan@zzm.uzh.ch

**Keywords:** at-home bleaching, hydrogen-peroxide, nano-hydroxyapatite

## Abstract

This study aimed to evaluate two hydrogen peroxide (HP)-based at-home bleaching systems in order to analyze whether nano-hydroxyapatite (nHA) addition may represent a reliable and safe solution for tooth whitening without altering dental microstructure and hardness. Human third molars (N = 15) were treated with two bleaching agents, one containing 6%HP (6HP) and the other 6% HP nHA-enriched (6HP-nHA) with average particle diameter ranging from 5–20 nm. Their effects on enamel were assessed using a spectrophotometer, Vickers microhardness (VMH) test and Scanning Electron Microscopy (SEM), comparing the treated groups with the non-treated control group (CTR). Color analysis revealed improvement in whiteness in both groups compared to CTR. VMH test results showed no differences among the groups. SEM analysis highlighted no evident changes in the enamel microstructure of tested groups compared to CTR. At high magnification, in 6HP group, a slight increase in irregularities of enamel surface morphology was observed, while 6HP-nHA group displayed removal of the aprismatic layer but preservation of the intact prismatic structure. These results suggest that the 6HP-nHA agent may be recommended to provide reliable whitening treatment, without damaging the enamel micromorphology and hardness.

## 1. Introduction

Dental bleaching procedures are often recommended to solve patient’s aesthetic demands for whiter teeth [1]. It is a well-accepted, noninvasive method for treating discolored teeth. Bleaching treatments consisting of the application of oxidizing agents, which directly break the double bonds of chromophores, correcting and reducing intrinsic and/or external discoloration resistant to mechanical cleaning [2]. The dental bleaching treatment can be performed by the dental clinician using bleaching agent with a high concentration of bleaching agent (in-office bleaching), or alternatively by the patient, dispensing a low concentration of bleaching agent into an individualized tray (at-home bleaching) [3]. Nevertheless, at-home bleaching has gained popularity among these types of bleaching treatments since it requires reduced chair-time, affords satisfactory outcomes, and causes less risk of tooth sensitivity than in-office treatment [4]. The 10% carbamide peroxide (CP) represents the gold standard for at-home bleaching treatment and has showed excellent bleaching results [4]. Considering that approximately 2–8 h/day per 2–3 weeks is required for CP to produce the desired results [5,6], these long sessions are perceived by patients as uncomfortable [5,7]. Several protocols using different bleaching agents have been proposed to overcome the issues associated with CP [8]. Among the potential bleaching agents used for at-home applications is the hydrogen peroxide (HP), which represents a reliable alternative to CP as it provides a faster release and greater efficiency, thus decreasing usage times to 30 min–1 h per day [9,10]. 

Despite its widespread use, there are potential severe side effects associated with the use of HP for at-home bleaching procedures, such as dentine hypersensitivity, alteration of tooth surface morphology and gingival irritation [11,12]. The addition of nano-hydroxyapatite (nHA) to the bleaching agents has been introduced as an attempt to address these problems. [13]. Indeed, the HA is the primary component of bone and teeth [14], and it is one of few materials that can facilitate the repairing or substituting of hard tissues [15]. Nano-HA refers to the HA particles of which particle size ranges from 8–39 nm [16], and the crystallite of natural HA in teeth revealed a high similarity to the synthetic nHA. nHA has a strong affinity for demineralized dental surface because of its ability to bind to pores produced due to bleaching treatment. After adhering to the tooth surface, nHA multiplies and organizes into microclusters to form a uniform apatite layer that may completely bond to interprismatic and prismatic enamel [17,18]. Despite this, the outcomes of nHA-enriched bleaching agent are still unknown. For instance, color and surface changes should be investigated in order to evaluate their efficacy [8]. The most frequently used method to evaluate the color is based on the International Commission on Illumination (Commission Internacional de l’Eclairage, CIE) LAB system [19]. CIELAB system is a color space which allows for evaluation of different color parameters. In order to evaluate the whitening effect of the bleaching agent, the most representative parameters are the b* and L* values [20,21]; the b* value highlights the color change from yellow to blue, which results in a perceivably whiter tooth, while the L* value describes the lightness of the surface. However, the b* value is mainly used for the blue-covarine based substances. CIELAB system also allows the calculation of color variation by using ΔE*_ab_, which quantifies the difference between two colors.

The investigation of hardness and morphological changes of enamel surface becomes important in the evaluation dental hard tissues’ preservation after bleaching procedures. Scanning Electron Microscopy (SEM) and a microhardness tester are used to evaluate possible morphological modifications and loss of mechanical properties, respectively, of surfaces of bleached teeth [22]. Enamel cannot be naturally repaired or replaced once completely lost, therefore its partial demineralization or superficial alteration should be avoided to ensure the preservation of a sound enamel structure [22,23]. The investigation of the morphological and hardness changes of enamel, as well as the achievement of its final color become fundamental to evaluate the efficacy and the safety of the bleaching treatments. To the best of our knowledge, although numerous protocols have considered different combinations of HP percentages, with or without the presence of nHA [11,22,24], there are no studies which compare the color changes and microstructural modifications of 6% HP bleaching agent with or without nHA when used for at-home bleaching. Therefore, the present in vitro investigation aims to evaluate whether 6% HP bleaching agent enriched with nHA could potentially be advantageous in obtaining an optimum whitening effect without causing any damage to the dental surface.

## 2. Materials and Methods

A total of 15 sound third molars were collected from subjects aged between 18 and 30 years. Teeth were surgically extracted for periodontal or orthodontic reasons. According to the Local Ethical Committee guidelines and the 1964 Helsinki declaration, informed consent was obtained from the subject aware that their hard-dental tissues, as discard of the surgical procedures, would have been used for research purposes. After the surgical extraction, teeth were washed in an ultrasonic bath with distilled water for 2 min in order to remove the blood and biological remains. Then, they were carefully examined to exclude the presence of lesions and decays, including hypoplastic defects and cracks [13]: teeth exhibiting any of these features were excluded. Selected teeth were stored in a 0.5% (*w/w*) chloramine solution (NH_2_Cl). Two days before the beginning of the study, samples were stored in distilled water at 37 °C for 24 h. Then, all samples were stored at room temperature in artificial saliva for 24 h consisting of: Na_2_PO_4_ 0.170 g, sodium ascorbate 0.001 g, glucose 0.015 g, NaCl 0.290 g, CaCl_2_ 0.085 g, NH_4_Cl 0.080 g, KCl 0.635 g, NaSCN 0.080 g, KH_2_PO_4_ 0.165 g and urea 0.100 g. The final pH was adjusted to 7. Two commercial at-home bleaching agents were tested: a commercially available bleaching agent composed of 6% hydrogen peroxide (6HP) (White Dental Beauty, Novon, Optident Optident, Ilkley, West Yorkshire, UK) and the other one containing 6% HP and nHA (6HP-nHA) (BioWhiten, Biodent Ltd., Istanbul, Turkey). White Dental Beauty consisted of one single syringe; on the other hand, BioWhiten products consisted of two parts, A and B, HP and nHA respectively. Following the manufacturer instructions, Part A and Part B were mixed together immediately before the application together to obtain a 1:3 mix of HP6/nHA. Details about the bleaching agents, as manufacturers, chemical composition and their use in terms of exposure times are reported in Table 1.

The samples were divided into three groups, each one consisting of five teeth: a control group (CTR), for comparison and then not submitted to any treatment; a 6HP group was bleached with White Dental Beauty; a 6HP-nHA group was bleached with BioWhiten. After rinsing the samples with distilled water, the bleaching agent was applied on the vestibular surface of the teeth, until the whole surface was covered. Between bleaching sessions, samples were stored at room temperature in artificial saliva in order to better simulate the clinical environmental.

### 2.1. Color Evaluation

The color of the samples was evaluated at the baseline (t0), as reference, before the bleaching application, and after 7 days of the bleaching application (t7). The color evaluations were performed by means of a spectrophotometer, the SpectroShade-Micro (MHT S.p.a., Verona, Italy). Before the color evaluation, each sample was rinsed in distilled water and during each measurement session, the spectrophotometer was calibrated according to the manufacturer’s recommendations by using the supplied white and green calibration standards. The measurements were established in mathematic coordinates referred to the international color space CIELAB: L* (lightness, where 100 represents white and 0 represents black), a* (red-green chromatic coordinate) and b* (blue-yellow chromatic coordinate). The measurements were taken by placing samples on not reflected black background (L = 9.20; a = 1.47; b = −3.83). The measurements were repeated three times for each specimen on the center of the vestibular dental surface, and the average values of L*, a*, and b* were calculated.

ΔE*_ab_ was calculated between the color at t0 and t7, using the following equation:

ΔE*_ab_ = [(L_t7_ *− L_t0_ *)^2^+ (a_t7_ *− a_t0_ *)^2^+ (b_t7_ *− b_t0_ *)^2^]^1/2^ (1)

Variation of L*, a*, and b* parameters were calculated by the following:

ΔL* = L_t7_ *− L_t0_ * (2)

Δa* = a_t7_ *− a_t0_ * (3)

Δb* = b_t7_ *− b_t0_ * (4)

### 2.2. Microhardness Evaluation

The Vickers microhardness (VMH) test was performed using a Remet microhardness tester HX-1000TM (Remet S.A.S., Casalecchio di Reno, Italy). In the center of the vestibular surface of each sample, specific areas were selected. Three indentations were performed on each area using a pyramid-shaped diamond indenter, loaded with 200 g for 15 s. Both diagonals of the imprint were measured by Proximo ver. 9 software, which calculates the average and subsequently the HV microhardness number, expressed as Kg/mm^2^.

### 2.3. Scanning Electron Microscopy Observations

Scanning Electron Microscopy (SEM) observations were carried out by a Zeiss Supra 40 field-emission electron microscope (Zeiss, Oberkochen, Germany). The same samples analyzed by VMH test were suitably placed in a sample holder and metallized with a vacuum precipitation of a gold film on the dental surface. SEM micrographs of the enamel were obtained with magnifications of 400×, 2000× and 8000×. SEM operated at 20 kV and at a 6 mm working distance. The obtained micrographs were used to evaluate the micromorphology of the dental enamel of all different analyzed tooth groups before and after the bleaching treatments.

### 2.4. Statistical Analysis

The homogeneity and normality of the data were evaluated. Significant differences between experimental groups were determined by means of ANOVA one-way test and two-way repeated measures ANOVA, followed by Tukey test, using the statistical software package Test Analysis of Excel (Office 365, Microsoft Corporation, Bellevue, WA, USA). Significance was set at *p* < 0.05. The sample size was calculated using Excel based on mean and standard deviation of the ΔL* value in a preliminary study.

## 3. Results

The color parameters of each group were described in Table 2.

Differences of single parameter and color were calculated and reported in Table 3. The lowest color variation between baseline t0 and t7 was achieved in CTR (ΔE*_ab_ = 5.8 ± 2.34). 6HP and 6HP-nHA showed the highest ΔE*_ab_ (14.09 ± 2.52 and 14.06 ± 2.90, respectively); however, they were not statistically different (*p* > 0.05). Regarding the single-color parameter, Δa showed no difference between all groups (*p* > 0.05). However, ΔL and Δb of 6HP and 6HP-nHA were statistically different than CTR (*p* < 0.05). Indeed, at t7, 6HP and 6HP-nHA presented L* values higher than CTR (6HP = 74.44 ± 3.31; 6HP-nHA = 75.88 ± 3.26; CTR = 67.59 ± 6.03).

**Figure 1:**
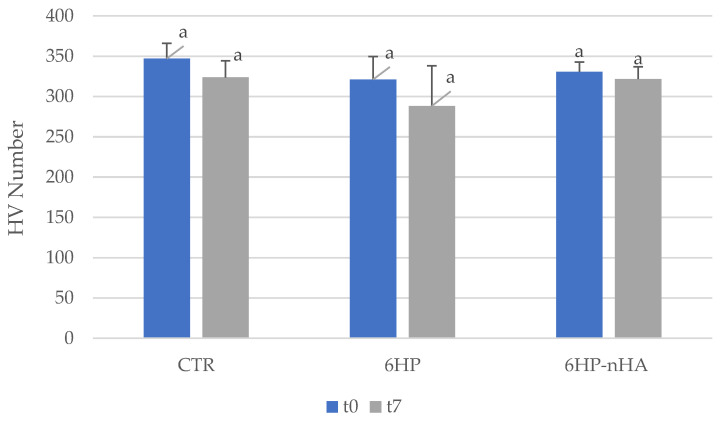
Microhardness values, expressed as HV number (Kg/mm^2^), of Control Group (CTR), 6% hydrogen peroxide (6HP) and 6% hydrogen peroxide and nano-hydroxyapatite (6HP- nHA) at the baseline (t0) and after 7 days after bleaching treatment (t7). Different superscript letters indicate statistical significance. HV—Hardness value. Two-way repeated measures ANOVA—Tukey’s multiple comparison test, *p* < 0.05.

Moreover, at t7, 6HP and 6HP-nHA showed b* values lower than CTR (6HP = 14.28 ± 2.35; 6HP-nHA = 11.25 ± 2.73; CTR = 17.57 ± 3.48). Regarding VMH measurements, CTR, 6HP and 6HP-nHA showed no statistically significant difference between t0 and t7 (Figure 1).

Although color and microhardness remained stable after the treatments with the tested bleaching agents, differences were detected on surface micrographs of 6HP and 6HP-nHA. 6HP showed an irregular surface and structure, as no rods neither interrods were detected. In contrast, in 6HP-nHA, the enamel structure, consisting of rods and interrods, was maintained.

**Figure 2:**
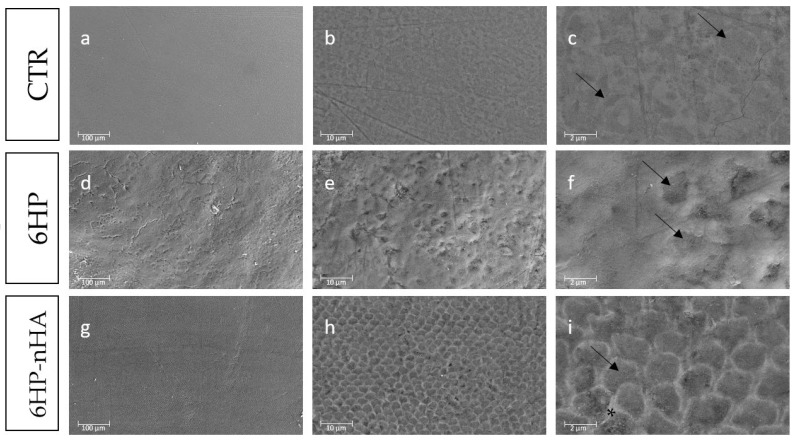
Scanning electron micrographs of a representative Control Group (CTR), 6% hydrogen peroxide (6HP) and 6% hydrogen peroxide enriched with nano-hydroxyapatite (6HP-nHA) at 400× (a,d,g), 2000× (**b**,**e**,**h**) and 8000× (**c**,**f**,**i**) original magnification. CTR: Micrographs (**a**) and (**b**) display the typical aspect of sound enamel morphology, with the aprismatic layer. In (**c**), the rods-like structure is slight visible (black arrows). 6HP: Micrograph (**d**) displays a partially sound enamel morphology with enamel-like crystal structure. In (**e**) loss of enamel surface integrity structure and an irregular surface are shown. Micrograph (**f**) shows the slight dissolution of prisms with partial destruction of the rods and interrods structure (black arrows), presenting a more irregular surface. 6HP-nHA: Micrograph (**g**) displays a sound enamel morphology with enamel-like crystals. In (**h**) and (**i**) the structure of the enamel prisms is emphasized since the rods and interrods are clearly distinguishable (black arrow and asterisk, respectively).

SEM analysis displayed different enamel surface alterations of the tested groups (Figure 2). Although at low magnification (400×), CTR showed only slight differences compared to the bleached groups, at higher magnification (2000× and 8000×) the differences among groups became more apparent. The CTR micrograph displays the typical sound enamel morphology, with an aprismatic superficial enamel layer, even at high magnification (Figure 2a–c). Conversely, the 6HP micrograph highlighted a minimal loss of integrity, with an increase in enamel irregularities (Figure 2d). Moreover, 6HP micrographs presented slight morphological surface alterations, with the presence of micro-porosities, an irregular surface pattern, with the partial dissolution of the rods and raised enamel interrods (Figure 2e,f). In Figure 2g, the 6HP-nHA micrograph displayed a sound and intact enamel surface similar to that of CTR, although the prismatic enamel layer was revealed. Moreover, at high magnification, 6HP-nHA micrographs showed prismatic features at the superficial layer, similar to those found in CTR, even if there was an evident increase of rods, presenting a honey-comb structure typical of the prismatic layer (Figure 2g–i).

## 4. Discussion

Tooth whitening treatment has become increasingly popular; several bleaching agents are available on the market with different protocols [25]. Patients often seek noninvasive bleaching treatment that offers the best results in the shortest time. Consequently, it is crucial to understand the characteristics of these bleaching products in order to achieve not only the best aesthetic results, but to achieve these results without compromising tooth structure. nHA is an adjunct to bleaching agents, which aids in limiting the side effects caused by tooth whitening. To date, only a few scientific articles are available about the potential beneficial effect of nHA enriched HP when used for at-home bleaching treatment [13,26,27].

Our results showed that the two tested groups obtained satisfactory results in color change when using the same protocol. The effectiveness of teeth whitening, indicated by the difference between the pretreatment and post-treatment tooth color, was determined by the ΔE*_ab_ values: 6HP and 6HP-nHA did not present a statistically significant difference (Table 3, *p* > 0.05). ΔE*_ab_ values of all groups were higher than the perceptibility threshold of 1.2 [28].

In accordance with Lilaj et al.’s study, the present results showed that bleaching products with a low concentration of HP and a short exposure time can be recommended for teeth whitening [29]. A possible explanation reported in the literature could be that, repeated short applications of low HP concentrations over a long period of time, resulted in whitening effects similar to those produced using high concentration products [30]. In a randomized clinical trial, Vano et al. analyzed the effectiveness, longevity and degree of hypersensitivity of whitened teeth using a low concentration of 6% HP with or without nHA [18]. In agreement with our study, both groups showed a significant improvement in tooth color, with no obvious differences, albeit the protocol used in the study was for in-office instead of at-home bleaching. Another in vitro report evaluated the color change and microhardness of enamel bleached with several agents, with or without nHA, concluding that the nHA does not alter the whitening effect of bleaching agents [31]. However, the authors used a higher concentration of HP and an in-office bleaching protocol.

Considering the whole color parameters, L* and b* play a crucial role in identifying the grade of tooth whitening [32]. L* value, which represents brightness, increased significantly in the two test groups, suggesting that regardless of the presence or absence of nHA, the two tested bleaching agents are effective at producing brighter teeth. ΔL between t0 and after 7 days reached values up to 10, with a value higher than the perceptual thresholds expressed by Paravina et al. [28].

Although b* is more suitable for the blue-covarine based substances, it still represents a whitening index, then it was discussed as well as L*. The b* value, which represents blue-yellow contrast, decreased significantly in the test groups after bleaching. In fact, 6HP and 6HP-nHA showed lower b* value (*p* < 0.05) than CTR (at t7, Table 2). The decrease in b* values at the end of treatment indicates a general improvement in the perception of the whiteness of the teeth [20,33]. As described in a previous study, the change in color from yellow to blue represents significant evidence of whitening, and the decrease of b* parameter is a crucial factor for the patient’s recognition of teeth whitening [21,34]. In fact, a change or shift to blue creates an appearance that visually appears whiter, changing the sharp color of the tooth towards white. Therefore, considering the three color parameters analyzed, it can be stated that there are no evident differences between the bleaching agents with or without nHA.

In addition to the color change evaluation, the VMH analysis and the morphological observations of the bleached enamel play a crucial role in understanding the effect of the applied treatment on the dental hard tissues. While SEM analysis allows a qualitative examination of the enamel microstructural changes at high magnification, the VMH test which relies on the reduction of mechanical properties, is a widely accepted method to determine the physical alterations of the enamel surface [35,36,37,38]. Although 6HP showed slightly lower average microhardness values than 6HP-nHA, there was no significant difference between the two groups, indicating that the microstructure of the enamel surface was not affected by the use of both tested bleaching agents. On the contrary, previous studies have observed that the use of HP resulted in morphological alterations of the enamel surface [25,30], due to the modification of the distribution of the enamel crystals [39] and the increase in porosity of the surface enamel [40]. It has been suggested that these enamel alterations are, in fact, due to the demineralization caused by the acidic effect of HP, while the reduction in microhardness is related to the combined effects of demineralization and destruction of the organic matter [41,42], especially when exposed to high concentrations of HP [43]. Nevertheless, few studies have investigated the adverse effects of bleaching agents using low HP concentrations [18,25,30].

Although the VMH test showed no differences between the bleaching agents with or without nHA, scanning electron micrographs highlighted qualitatively different alterations of the enamel surface between the CTR and tested groups. 6HP has revealed a slight increase in the enamel irregularities, with a partial loss of the interprismatic spaces and some microporosities. Instead, 6HP-nHA displayed a removal of the aprismatic layer and an evident presence of honeycomb structure, typical of the prismatic enamel layer [44], whilst still maintaining a sound structure without destruction of the rods. Therefore, it can be affirmed that the presence of nHA prevents an alteration of the enamel structure. These findings are consistent with the study by Bistey et al., which stated that the bleaching agents based on low HP concentration, without nHA, can lead to morphological alterations on the enamel surface [45]. In fact, nHA played a key role in maintaining the structure of the enamel during the bleaching treatment [45,46]. Indeed, HA is an alkaline salt, which increases the pH of HP solution from approximately 3.2 to around 5.4, therefore making it less acidic [46]. In addition, nHA particles may adhere evenly to the enamel surface, forming a protective layer for the underlying enamel, which would lessen the direct contact of HP with the enamel surface [47]. However, also the dimension of nHA plays a crucial role in their capability to react to enamel [48]. The nHA of the tested bleaching agent possess a rod-like morphology with precise dimensions expressed in Table 1. Indeed, nHA with dimension of 10–20 nm may promote the penetration of crystals into the interprismatic space through ion transportation or linking to the interprismatic protein, resulting in remineralization enamel surface [48,49]. All these effects exerted by nHA could lead to a great reduction in the enamel demineralization caused by HP [47]. 

Some authors have concluded on the contrary, that bleaching agent containing 15% HP produced a similar topography to that obtained for the non-bleached specimens [50], however different timings and magnifications used might have led to different results. A limitation of our study is that we have only evaluated the color with CIELAB and used only one bleaching protocol, without considering other variables related to the composition of the bleaching agents [51]. For this reason, further studies will be planned to investigate additional bleaching protocols with or without nHA adjunct, with an aim to reduce the treatment time for at-home bleaching procedures, whilst still maintaining an intact structure of dental hard tissues.

## 5. Conclusions

The results obtained by the spectrophotometer showed that the application of bleaching gels based on 6% HP and 6% HP nHA-enriched enables the maintenance of the enamel microstructure, without influencing their color change capability. However, 6% HP bleaching gels enriched with nHA may confer an additional protective effect to enamel and therefore represent a safe and effective at-home bleaching system.

## Figures and Tables

**Table 1:** Details of the used bleaching agents.

Experimental Group	Manufacturer	Chemical Composition	Exposure Time
**6HP**	White Dental Beauty, Novon, Optident Ilkley, West Yorkshire, UK	6% hydrogen peroxide	50 min/day for 7 days
**6HP-nHA**	BioWhiten, Biodent Ltd., Istanbul, Turkey	water, glycerin, alcohol, sodium bicarbonate, sodium hydroxide, 6% hydrogen peroxide and nano hydroxyapatite * (ratio 1:3)	50 min/day for 7 days

* Rod-like morphology, width 5–20 nm (typically close to 10 nm) and length < 50 nm (typically between 20 to 40 nm).

**Table 2:** CIELAB parameters of Control Group (CTR), 6% hydrogen peroxide (6HP) and 6% hydrogen peroxide and nano-hydroxyapatite (6HP- nHA) at the baseline (t0) and after 7 days after bleaching treatment (t7).

	CTR		6HP		6HP-nHA	
	t0	t7	t0	t7	t0	t7
L*	65.60 ± 6.59	67.59 ± 6.03	64.07 ± 2.88	74.44 ± 3.31	64.91 ± 2.85	75.88 ± 3.26
a*	3.56 ± 2.26	3.76 ± 2.02	3.62 ± 0.39	1.74 ± 0.96	2.73 ± 1.17	1.28 ± 0.65
b*	20.83 ± 3.00	17.57 ± 3.48	22.69 ± 2.10	14.29 ± 2.35	19.02 ± 3.49	11.25 ± 2.73

**Table 3:** Average and standard deviation of color differences (ΔE*_ab_) and single parameter differences (ΔL*, Δa*, Δb*) between the baseline and after 7 days of Control Group (CTR), 6% hydrogen peroxide (6HP) and 6% hydrogen peroxide and nano-hydroxyapatite (6HP- nHA). Different superscript letters indicate statistical significance between groups. One-way ANOVA—Tukey’s multiple comparison test, *p* < 0.05.

	CTR	6HP	6HP-nHA
ΔE*_ab_	5.80 ± 2.34 ^a^	14.09 ± 2.52 ^b^	14.06 ± 2.90 ^b^
**ΔL** *	2.21 ± 3.69 ^a^	10.37 ± 2.52 ^b^	10.97± 3.48 ^b^
Δa*	0.20 ± 1.03 ^a^	−1.88 ± 1.05 ^a^	−1.45 ± 0.82 ^a^
Δb*	−2.78 ± 3.70 ^a^	−8.4 ± 2.47 ^b^	−7.63 ± 2.76 ^b^

## Data Availability

The data presented in this study are available on request from the corresponding author G.O.
